# Integrative analysis reveals CD38 as a therapeutic target for plasma cell-rich pre-disease and established rheumatoid arthritis and systemic lupus erythematosus

**DOI:** 10.1186/s13075-018-1578-z

**Published:** 2018-05-02

**Authors:** Suzanne Cole, Alice Walsh, Xuefeng Yin, Mihir D. Wechalekar, Malcolm D. Smith, Susanna M. Proudman, Douglas J. Veale, Ursula Fearon, Costantino Pitzalis, Frances Humby, Michele Bombardieri, Amy Axel, Homer Adams, Christopher Chiu, Michael Sharp, John Alvarez, Ian Anderson, Loui Madakamutil, Sunil Nagpal, Yanxia Guo

**Affiliations:** 1 Immunology, Janssen Research, 1400 McKean Road, Spring House, PA 19477 USA; 2 Rheumatology Unit, Repatriation General Hospital and Flinders University, Adelaide, Australia; 3 Rheumatology Unit, Royal Adelaide Hospital, Adelaide, Australia and Discipline of Medicine, University of Adelaide, Adelaide, Australia; 4 Center for Arthritis and Rheuamatic Diseases, St. Vincent’s University Hospital, University College Dublin, Elm Park, Dublin 4, Ireland; 5 Molecular Rheumatology, Trinity Biomedical Sciences Institute, Trinity College Dublin, Trinity College London, Dublin 24, Ireland; 6 Queen Mary University of London, Charterhouse Square, London, EC1M 6BQ UK; 7 Oncology, Janssen Research, 1400 McKean Road, Spring House, PA 19477 USA

**Keywords:** CD38, Plasma cell, Daratumumab, Rheumatoid arthritis, Systemic lupus erythematosus

## Abstract

**Background:**

Plasmablasts and plasma cells play a key role in many autoimmune diseases, such as rheumatoid arthritis (RA) and systemic lupus erythematosus (SLE). This study was undertaken to evaluate the potential of targeting CD38 as a plasma cell/plasmablast depletion mechanism by daratumumab in the treatment of patients with RA and SLE.

**Methods:**

RNA-sequencing analysis of synovial biopsies from various stages of RA disease progression, flow cytometry analysis of peripheral blood mononuclear cells (PBMC) from patients with RA or SLE and healthy donors, immunohistochemistry assessment (IHC) of synovial biopsies from patients with early RA, and ex vivo immune cell depletion assays using daratumumab (an anti-CD38 monoclonal antibody) were used to assess CD38 as a therapeutic target.

**Results:**

We demonstrated that the plasma cell/plasmablast-related genes *CD38*, *XBP1*, *IRF4*, *PRDM1*, *IGJ* and *TNFSF13B* are significantly up-regulated in synovial biopsies from patients with arthralgia, undifferentiated arthritis (UA), early RA and established RA as compared to healthy controls and control patients with osteoarthritis. In addition, the highest CD38 expression was observed on plasma cells and plasmablasts compared to natural killer (NK) cells, classical dendritic cells (DCs), plasmacytoid DCs (pDCs) and T cells, in blood from healthy controls and patients with SLE and RA. Furthermore, IHC showed CD38 staining in the same region as CD3 and CD138 staining in synovial tissue biopsies from patients with early RA. Most importantly, our data show for the first time that daratumumab effectively depletes plasma cells/plasmablasts in PBMC from patients with SLE and RA in a dose-dependent manner ex vivo.

**Conclusion:**

These results indicate that CD38 may be a potential target for RA disease interception and daratumumab should be evaluated clinically for the treatment of both RA and SLE.

**Electronic supplementary material:**

The online version of this article (10.1186/s13075-018-1578-z) contains supplementary material, which is available to authorized users.

## Background

Autoantibodies play an important role in the pathogenesis of many autoimmune diseases, including rheumatoid arthritis (RA) [1] and systemic lupus erythematosus (SLE) [2]. Classical autoantibodies target the tissue directly or via formation of immune complexes [3]. Short-lived plasmablasts and plasma cells and long-lived plasma cells can generate high titers of autoantibodies upon activation. Long-lived plasma cells reside in bone marrow and inflammatory tissue niches and produce copious amounts of autoantibodies independent of B cell activation. Studies have shown an increased number of plasmablasts or ratio of plasmablast/B cells in the blood of patients with active SLE [4]. Although the bone marrow provides the survival niche for long-lived plasma cells [5, 6], inflammatory tissues bear high B cell-activating factor (BAFF) and a proliferation-inducing ligand (APRIL), maintain long-lived plasma cell survival, and thus contribute to the autoantibody secretion in inflammatory joints in patients with RA [7] and nephritic kidneys in NZB/W mice [8]. Different therapeutic agents have been developed to target antibody production in autoimmune diseases. Rituximab (anti-CD20) and belimumab (anti-BLyS) prevent short-lived plasmablasts from proliferating and reduce autoantibody production to some degree. However, non-proliferative long-lived plasma cells are not susceptible to this effect. Short-term treatment with the proteasome inhibitor bortezomib followed by B-cell target therapy (anti-CD20) decreases anti-dsDNA-secreting plasma cells and delays the development of nephritis in NZB/W mice [9]. Clinical studies show that bortezomib depletes plasma cells and ameliorates disease in patients with refractory SLE [10, 11]. Inhibition of plasma cell survival via blockade of BAFF and APRIL has also been tested. Atacicept (soluble TACI-Ig) has shown no clinical response in patients with RA [12]. Though atacicept at a lower dose (75 mg) was proved non-effective in prevention of flares in SLE in patients with moderate to severe disease, a higher dose (150 mg) proved beneficial on flare rate and time to first flare. Unfortunately, the trial was terminated due to two deaths caused by pneumonia in that study group [12]. Aside from the strategies mentioned above, it is worthwhile to determine if plasma-cell-specific targets may prove more efficacious in the treatment of autoimmune diseases, such as RA and SLE.

CD38 is a type II glycoprotein highly expressed on plasma cells, memory B cells and multiple myeloma (MM) cells [13]. Daratumumab, a depleting monoclonal anti-CD38 antibody, has been approved for the treatment of patients with MM [14, 15]. Plasma cell depletion in the bone marrow has been observed in patients with MM treated with daratumumab [16]. It is therefore reasonable to hypothesize that CD38 can be a target for the treatment of autoimmune diseases by depleting plasma cells specifically. Previous studies have shown CD38 expression in the synovial biopsies of patients with established RA [17]. A comprehensive analysis of CD38 expression on various immune cells in patients with autoimmune diseases could facilitate the dosing possibility of depleting only plasmablasts and plasma cells without affecting other potentially beneficial cells. Furthermore, despite the fact that anti-citrullinated protein antibodies (ACPA) may already exist in pre-RA stages, no studies have analyzed the dynamic role that plasma cells and plasmablasts may play during RA disease progression, namely from arthralgia to undifferentiated arthritis (UA), early RA and established RA. It is possible that targeting CD38 at the pre-disease stage could achieve greater disease suppression in patients with RA and SLE. Therefore, in this study, we investigated the potential of targeting CD38 at various stages of RA progression. Most importantly, for the first time, we evaluated the efficacy of a U.S. Food and Drug Administration-approved cancer drug targeting CD38, daratumumab, in depleting plasma cells/plasmablasts ex vivo in PBMC from patients with RA and SLE, to provide a rationale for clinical trials of daratumumab in patients with RA and SLE.

Herein, RNA sequencing (RNA-Seq) showed that various plasma cell/plasmablast-related genes are significantly up-regulated in pre-disease (arthralgia and UA) and RA (early and established) synovial tissue biopsies compared to counterpart healthy donors or patients with osteoarthritis (OA). Furthermore, the highest CD38 expression in peripheral blood was observed on plasma cells and plasmablasts, followed by NK cells, pDCs, a regulatory T cell (Treg) subpopulation and naïve T cells in healthy donors and patients with SLE and with RA. Immunohistochemistry assessment showed the presence of plasma cells and T cells in the synovial biopsies from patients with early RA. Finally, we demonstrated that daratumumab depletes plasma cells/plasmablasts in a dose-dependent manner ex vivo.

## Methods

### Human subjects

The human donors (healthy controls and people with OA, arthralgia, UA, early RA and established RA) in this study have been described previously [18]. Briefly, synovial biopsy specimens from healthy and donors with OA were obtained from a group of patients attending a sports medicine facility with knee pain. Healthy subjects were defined as those who had no arthritis, cartilage damage or synovitis on knee arthroscopy. Donors with OA had clinical history and/or examination findings suggestive of OA in addition to supportive arthroscopic findings. The average age of healthy donors was 35.2 years. The average age of donors with OA was 49.0 years. There were 13 women (59.1%) and 9 men (40.9%). Patients with arthralgia (*n* = 10) were subjects with symptoms of aches and pains, without clinical signs of synovitis or significantly raised C-reactive protein (CRP) (mean = 4.49 mg/l) at first assessment, but with positive circulating rheumatoid factor (RF^+^) and ACPA. There were eight women and two men, and the mean age was 51.6 years (range 34–66 years). Patients with undifferentiated arthritis/inflammatory arthritis (UA/IA) (*n* = 6) were defined as subjects presenting with clinical signs of synovitis, but who did not meet the 2010 American College of Rheumatology (ACR) criteria for RA. All six patients were female, the mean age was 46 years, and they had significantly raised CRP (mea*n* = 17.66 mg/l). Early RA (*n* = 57) was defined as within 12 months of disease diagnosis without prior small or large molecule disease modifying anti-rheumatic drugs (DMARDs) usage (mean disease duratio*n* = 5 months). Early RA synovial tissue biopsies were collected from two different cohorts of patients identified at Flinders University and Queen Mary University of London. There were 33 women and 22 men, and the mean age was 55.9 years. Established RA (*n* = 95; disease duration >1 year) synovial tissue biopsies were collected from two different cohorts of patients identified at Queen Mary University of London and St. Vincent’s Hospital. The average disease duration in this patient population was 68 months. The average age in the group was 54.0 years. All patients with established RA had received small molecule DMARDs or anti-TNFα treatments. More details about the clinical characteristics of these subjects included in this analysis can be found in a report of a previous study [18].

PBMC samples for CD38 expression analysis from patients with SLE and RA and from healthy donors were acquired from Bioreclamation (Westbury, NY, USA) and Precision for Medicine (Frederick, MD, USA). All donors with SLE were clinically active at the most recent visit into the clinic before the blood draw and under standard of care treatments, including prednisone, benlysta (*n* = 1), rituximab (*n* = 1) and other small molecule therapeutics. One patient was treatment-naïve at the time of the blood draw. The mean SLE disease activity index (SLEDAI) scores in these subjects was 18.7 (range 6–32), which indicates active disease. All donors with RA were clinically active at the time of blood draw and had received various DMARD treatments. The biologic treatments included Orencia (*n* = 1), Simponi (*n* = 1), Simponi Aria (*n* = 1), Humira (*n* = 1), and Enbrel (*n* = 3). All subjects had received small molecule DMARD treatments before the blood draw.

All protocols for collecting synovial biopsies and blood/serum were approved by the Institutional Review Board. All patients signed the consent form for participating in the study.

### RNA-Seq gene expression analysis

RNA-Seq analysis was performed on the same synovial biopsies using the same algorithm as reported previously and the RNA-Seq data were deposited in the Gene Expression Omnibus (GEO) database as part of a previous study [GEO:GSE89408] [18].

Total RNA was extracted from frozen synovial biopsies from patients with RA and only RNA samples that passed quality control by an Agilent Bioanalyzer were further analyzed. RNA-Seq was performed by Q2 Solutions (Morrisville, NC, USA). Sequencing libraries were prepared using TruSeq Stranded Total RNA RiboZero protocol from Illumina. Libraries were pooled and sequenced with an Illumina HiSeq 2000 with paired-end 100-bp flow cells. FastQC was used to evaluate raw read quality.

Reads were trimmed for adaptors and sequence quality. The average number of clusters (post-trimming) per sample was 8.9 × 10^7^. Trimmed reads were aligned to the human b37.3 reference genome using the STAR v2.4 aligner [19]. Aligned reads were quantified using RSEM v1.2.14 algorithm [20] with UCSC transcriptome model (accessed on 17 March 2014) that included long intergenic non-coding RNAs (lincRNAs) from Ensembl v75. This transcriptome model has a total of 34,495 genes and 88,933 isoforms. Aligned data were evaluated for quality using several metrics (e.g., mapping rate, coverage) and visually inspected for deviation from the population across multiple metrics and principal components analysis. Statistical testing of RNA-Seq data was performed in R with the “limma” package [21]. Counts were converted to log2 counts per million, quantile normalized and precision weighted. A linear model was fitted to each gene, and empirical Bayes moderated *t* statistics were used to assess differences in expression.

### Fluorescence-activated cell sorting (FACS) analysis

PBMC samples were analyzed in three different staining panels for CD38 expression as follows: Panel 1: CD38-FITC, CD14-PE, HLA-DR-PerCPCy5.5, CD11b-PECy7, CD33-APC, BDCA2-VioBlue, CD16-BV510, Lineage (CD3/CD8/CD4/CD19)-BV605, CD45-BV650, CD11c-BV711 and CD56-BV786. Panel 2: CD38-FITC, CD62L-PE, CCR7-PerCPCy5.5, CD27-PECy7, CD4-APC, CD127-BV421, CD8-BV510, CD3-BV605, CD25-BV650 and CD45RA-BV786. Panel 3: CD38-FITC, BCMA-PE, CD24-PerCPCy5.5, IgD-PECy7, CD20-APC, CD27-BV421, IgM-BV510, CD138-BV605, CD3-BV650, CD56-BV650 and CD19-BV711. For the ex vivo depletion assay, a different panel was used to measure NK cells and plasma cells/plasmablast in one panel as follows. Panel: CD38-FITC, CD138-PE, IgD-PECy7, CD20-APC, Live-Dead/Near-IR, CD27-Pacific Blue, CD3-BV605, CD56-BV650, and CD19-BV711. All antibodies were purchased from BD Bioscience except for the following: CD27-BV421, CD138-PE, CD56-BV650, BCMA-PE (Biolegend) and BDCA2-VioBlue (Miltenyi). For the analysis of CD38 expression on PBMC, CD38-FITC (Catalog number: CYT-38F2) was purchased from Cytognos (Salamanca, Spain). In the depletion assay, CD38 expression was analyzed using HuMax-003-FITC (Genmab/Janssen R&D), a monoclonal antibody (Ab) that binds to a different epitope than daratumumab, as described previously [22]. Isotype controls and/or FMOs (Fluorescence Minus One) were used to determine gating boundaries. Samples were acquired using the LSRII (BD Bioscience) and analyzed using FlowJo software (Treestar Inc.).

### Immunohistochemistry analysis (IHC)

Frozen sections of synovial biopsies were in optimal cutting temperature compound and taken at 4-μm thickness for evaluation of protein markers in the samples by IHC. Tissue sections were briefly fixed in methanol, air dried and incubated with either anti-CD38 (SP149) (Cell Marque, Rocklin, CA, USA), anti-CD3 (2GV6) (Ventana, Tuscon, AZ, USA) or anti-CD138 (B-A38) (Abcam, Cambridge, MA, USA). The markers were visualized with 3,3′-diaminobenzidine and the relative expression of each marker was noted by JA and MS while blinded to the specimen group information. The scoring paradigm describes the observed expression of each marker as either abundant, moderate or low as determined by the number of positive cells counted within a total magnification of × 100 across the entire biopsy section. Abundant denotes strongly positive clusters of more than 20 cells in any field, moderate presence if between 5 and 20 and low if fewer than 5 cells were observed per field. The observation in the highest density field in a sample was recorded as the score. A pathologist, JA, reviewed indicated areas of inflammation and hyperplasia.

### Daratumumab depletion assay

PBMC samples from healthy controls and patients with RA or SLE were thawed in complete Roswell Park Memorial Institute medium (RPMI) and rested overnight before the assay. Cells were then cultured in 96-well U-bottom plates at 2.5 × 10^5^ cells/well. Human serum (Complement Technology, TX, USA) was applied at 10% to the culture as a source of complement. Daratumumab was added to the wells at concentrations indicated in the text. Isotype control, IgG1-b12 (Genmab), a human monoclonal Ab against an innocuous antigen (HIV1-gp120) [22], was applied at 1 μg/ml. Cells were cultured for 72 h before FACS, quantitative (q)PCR or branced DNA (bDNA) analysis. CountBright absolute counting beads (Life Technologies, CA, USA) were added to the samples before acquisition on A flow cytometer for quantification of each cell population. RNA was prepared from the same culture for qPCR analysis of immunoglobulin J (*IGJ*).

### RNA*,* qPCR and bDNA assay

RNA was prepared using Qiagen RNeasy kits (Qiagen) for qPCR analysis of *IGJ*. Complementary DNA (cDNA) was prepared using SuperScript IV VILO MasterMix and TaqMan qPCR assay with TaqMan Universal PCR Master Mix and samples were run on the VIIA7 instrument (Applied Biosystems). Primers include *IGJ* (Hs00376160_m1) and housekeeping genes *IPO8* (Hs00183533_m1) and *GUSB* (4332655). All cDNA and TaqMan reagents were purchased from Life Technologies (CA, USA).

Alternatively, a bDNA assay was used to measure *IGJ* messenger RNA (mRNA). After incubation, cells were pelletted and triplicate wells were pooled in 100 μl PBS and then pelleted again. Following the QuantiGene sample processing protocol for PBMC, each well was resuspended in 200 μl working lysis buffer and incubated for 30 min at 50–55 °C. Cell lysates were then stored at − 80 °C until needed for assay. A custom Quantigene Plex assay containing endogenous controls *TBP* and *HPRT1* and the target gene *IGJ* was run according to the manufacture’s manual and samples were analyzed on a Luminex 200 instrument (BioRad). For the fold-change (FC) of *IGJ* mRNA in the presence of daratumumab, the relative expression level of *IGJ* was normalized to *IGJ* mRNA in the isotype control group in some analyses.

### Statistics

For high-dimensional RNA-Seq, features were considered differentially expressed if they satisfied a 1.5-fold change and 0.05 adjusted *p* value cutoff unless otherwise specified. The Benjamini-Hochberg method was used to calculate *p* values adjusted for multiple hypotheses. Fold changes were calculated from log2(FC) estimates and reported with a positive sign for ratios greater than 1 (log2(FC) >0) and with a negative sign for ratios less than 1 (log2(FC) <0). Data from flow cytometry phenotyping and depletion experiments were analyzed using GraphPad™ Prism software (v7). One way analysis of variance (ANOVA) with Tukey’s test for multiple comparisons was performed to compare cohorts and cell types.

## Results

### The expression of plasma cell/plasmablast differentiation and survival-related genes is increased in synovial biopsies from various stages during RA progression

**Fig. 1:**
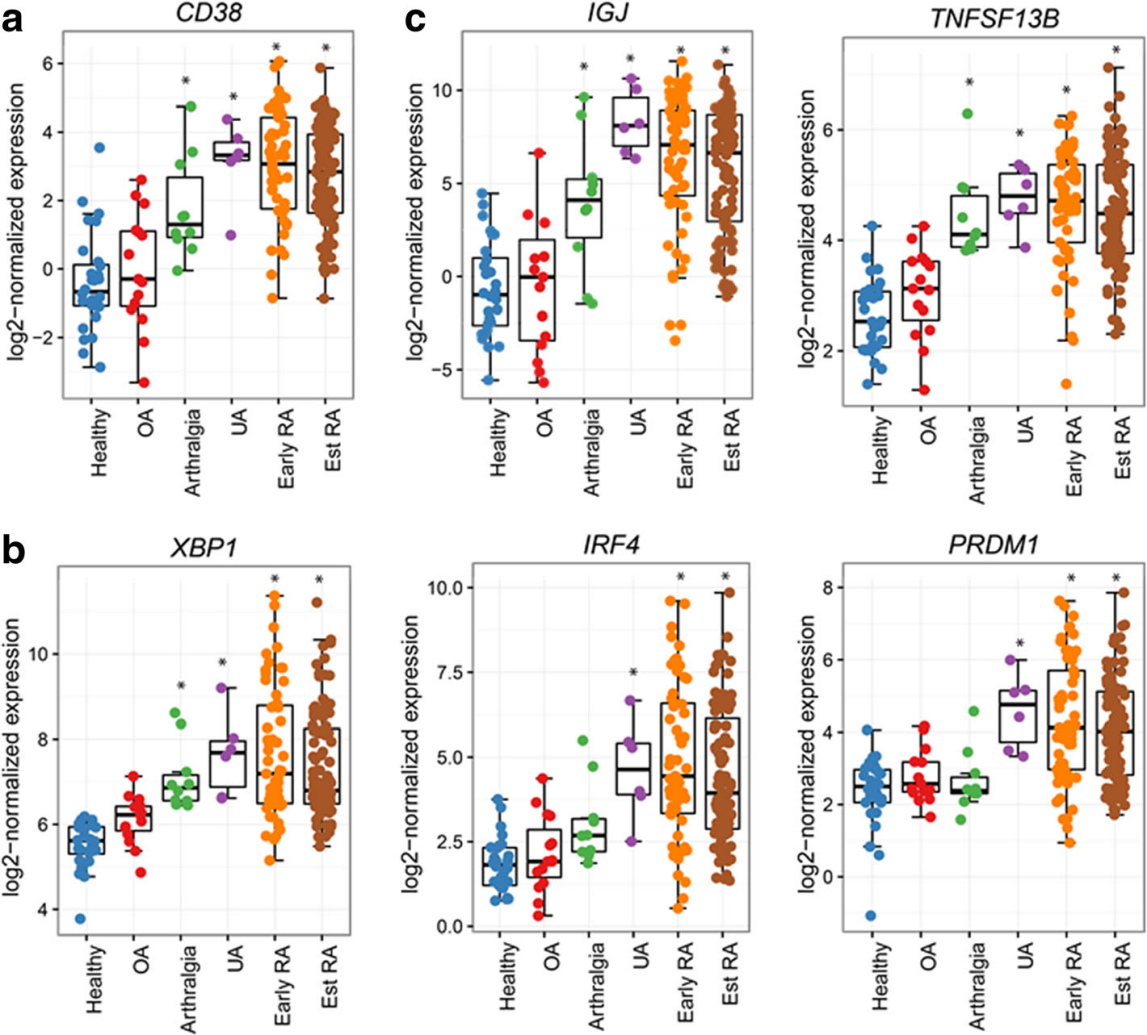
Plasma cell/plasmablast-related gene expression is increased at different stages of disease progression to rheumatoid arthritis (RA). RNA-sequencing was performed on biopsies from healthy tissue, and tissue from patients with osteoarthritis (OA), arthralgia, undifferentiated arthritis (UA), early RA and established RA (Est RA). **a** *CD38* expression stratified by disease group/stage. **b** Transcription factors *XBP1*, *IRF4*, and *PRDM1* mRNA were measured in synovial biopsies from different disease groups. **c** Plasma cell/plasmablast survival-related genes *IGJ* and *TNFSF13B* expression in synovial biopsies from different disease groups. *Statistically significant difference compared to healthy tissue (all graphs) (adjusted *P* < 0.05)

The involvement of CD38 and plasma cell/plasmablast-related genes in RA disease progression was evaluated by obtaining synovial tissue biopsies from patients with arthralgia, UA and early and established RA. Transcriptomics analysis by RNA-Seq in synovial tissue biopsies from healthy donors (*n* = 28) and patients with OA (*n* = 15), ACPA^+^ rheumatoid factor (RF)^+^ arthralgia (*n* = 10), UA (*n* = 6), early RA (*n* = 57), and established RA (*n* = 95) revealed significantly higher *CD38* expression as early as the arthralgia stage (compared to healthy controls, fold change (FC) = 4.5, *P* = 2.50E-04, Fig. 1a, Table 1) prior to established RA development. This trend continued in the patients with early (FC = 11.2, *P* = 3.11E-8) and established RA (FC = 8.5, *P* = 3.03E-17) (Fig. 1a, Table 1). Since specific transcription factors are essential for plasma cell differentiation, high antibody production and plasma cell homing to the bone marrow, we next examined the expression of X-box binding protein 1 (XBP1), interferon regulatory factor 4 (IRF4) and PR domain zinc finger protein 1 (PRDM1). As predicted, this analysis showed significantly higher expression of *XBP1*, *IRF4* and *PRDM1* in synovial tissues from patients with UA, early RA and established RA compared to patients with OA and healthy controls (Fig. 1b). In addition, *XBP1* was also significantly upregulated in arthralgia samples (FC = 2.97, *P* = 1.04E-03). Furthermore, as IgJ is highly enriched in plasmablasts and plasma cells [23] while BAFF (also known as TNFSF13B) is important for plasma cell survival [24] we also analyzed their expression. The data demonstrated that both *IGJ* and *TNFSF13B* are up-regulated in synovial biopsies from all stages of the disease, including arthralgia, UA, early RA and established RA (Fig. 1c and Table 1). Taken together, these data show increased expression of plasma cell/plasmablast differentiation and survival-related genes, indicating the involvement of these cells in the progression from asymptomatic arthralgia to established RA.

**Table 1:** Fold change and adjusted *P* values for comparison of genes compared in tissues from patients with disease and from healthy controls (see Fig. 1)

	*CD38*		*IGJ*		*TNFSF13B*		*XBP1*		*IRF4*		*PRDM1*	
Groups	Fold change	Adj. *p*	Fold change	Adj. *p*	Fold change	Adj. *p*	Fold change	Adj. *p*	Fold change	Adj*. p*	Fold change	Adj. *p*
OA-healthy	1.31	0.76	1.17	0.95	1.34	0.54	1.50	0.49	1.23	0.86	1.36	0.68
Arthralgia-healthy	4.55	2.50E-04	24.52	5.34E-04	3.48	2.27E-06	2.97	1.04E-03	2.30	0.11	1.23	0.62
UA-healthy	11.84	3.10E-06	516.33	1.86E-07	4.40	1.00E-05	4.35	4.45E-04	6.96	2.74E-03	4.75	1.18E-03
Early RA-healthy	11.20	3.11E-18	123.34	2.02E-15	3.95	1.79E-15	4.44	2.93E-12	8.42	7.15E-11	3.99	8.37E-09
Est. RA-healthy	8.50	3.03E-17	81.75	3.15E-15	3.83	6.63E-17	3.62	5.14E-11	6.33	8.30E-10	3.31	6.15E-08

*OA* osteoarthrits, *UA* undifferentiated arthritis, *RA* rheumatoid arthritis, *Est.* established, *Adj.* adjusted

### Plasma cells are present at higher frequency in PBMC from patients with SLE than from patients with RA and healthy donors

**Fig. 2:**
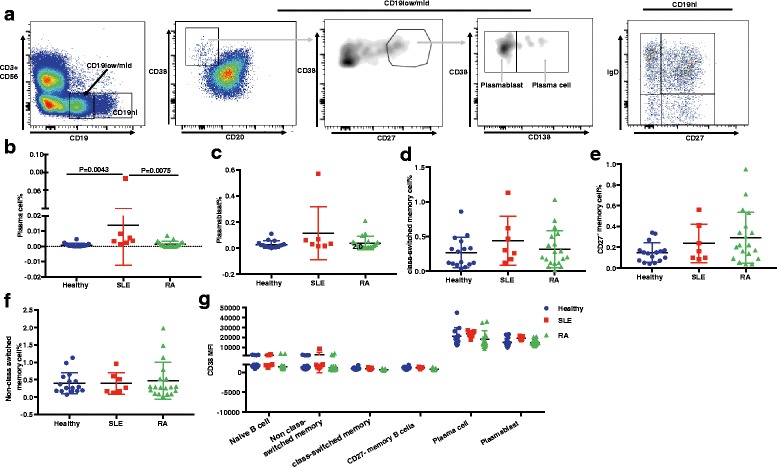
CD38 expression on immune cells in peripheral blood mononuclear cells (PBMC) from healthy donors and patients with systemic lupus erythematosus (SLE) or rheumatoid arthritis (RA). **a** Gating strategy for plasma cells and plasmablasts in PBMC samples. Plasma cells are gated as live singlet lymphocytes, CD3^−^CD56^−^CD19^low/mid^CD20^−^CD38^hi^CD27^hi^CD138^+^. Plasmablasts are gated as live singlet lymphocytes, CD3^−^CD56^−^CD19^low/mid^CD20^−^CD38^hi^CD27^hi^CD138^−^. CD19^hi^ B cells are separated into naïve B cell (IgD^+^CD27^−^), non-class-switched memory B cell (CD27^+^IgD^+^), class-switched memory B cell (CD27^+^IgD^−^) and CD27^−^ memory B cell (CD27^−^IgD^−^). **b** Percentage of CD27^hi^CD38^hi^CD138^+^ plasma cells in total lymphocytes from PBMCs from healthy controls and patients with SLE or RA. **c** Percentage of CD27^hi^CD38^hi^CD138^−^ plasmablasts in total lymphocytes from PBMCs from different groups. **d-f** Percentage of class-switched memory cell (**d**), CD27^−^ memory B cell (**e**), and non-class-switched memory B cell (**f**) in total lymphocytes. **g** Quantification of CD38 MFI in naïve B cell, non-class switched memory cell, class-switched memory cell, CD27^−^ memory cell, plasma cells and plasmablasts

**Fig. 3:**
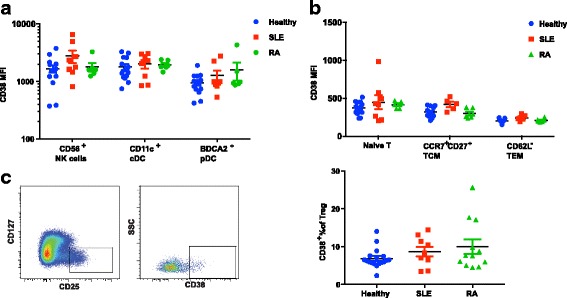
CD38 expression on myeloid, natural killer (NK) and T cells in peripheral blood mononuclear cells (PBMC) from healthy donors and patients with systemic lupus erythematosus (SLE) and rheumatoid arthritis (RA). **a** Quantification of CD38 MFI on CD56^+^CD16^+^ NK cells, CD11c^+^ classical DC, and BDCA2^+^ plasmacytoid dendritic cells (DC). **b** Quantification of CD38 MFI on CD45RA^+^ naïve T cells, CD62L^hi^CD27^hi^CCR7^+^CD45RA^−^ central memory T cells (T_CM_) and CD62L^−^CD45RA^−^ effector memory T cell (T_EM_). **c** Representative fluorescence-activated cell sorting (FACS) plot of CD38 expression on CD4^+^CD127^low^CD25^hi^ T regulatory cells (Tregs). **d** Proportion of the CD38^bri^ subset in peripheral Tregs from healthy controls and donors with SLE or RA

To evaluate the possibility of specifically targeting CD38 and plasma cells in patients with autoimmune disease, we evaluated the expression of CD38 across a spectrum of immune cell subpopulations in human PBMC samples. We used CD56 to gate out NK cells and CD3 to gate out T cells in the lymphocyte gate, and focused on CD19^low/mid^ to analyze plasma cells and plasmablasts. As shown in Fig. 2a, plasma cells are defined as CD3^−^CD56^−^CD19^low/mid^CD20^−^CD27^hi^CD38^hi^CD138^+^, whereas plasmablasts are CD3^−^CD56^−^CD19^low/mid^CD20^−^CD27^hi^CD38^hi^CD138^−^ lymphocytes. Detailed analysis shows that the proportion of plasma cells among lymphocytes are significantly elevated in SLE patients compared with healthy controls and patients with RA (Fig. 2a and b), consistent with previous studies [25]. A similar proportion of plasmablasts is observed among healthy donors and those with SLE or RA (Fig. 2a and c). We further used IgD and CD27 to analyze CD19^hi^ B cells, namely class-switched memory B cells (CD27^+^IgD^−^), non-class-switched memory B cells (CD27^+^IgD^+^), CD27^−^ memory B cells (CD27^−^IgD^−^) and naïve B cells (CD27^−^IgD^+^) (Fig. 2a, right panel). The proportion of class-switched memory cells, non-class-switched memory cells and CD27^−^ memory B cells was similar among healthy donors and patients with SLE or RA (Fig. 2d-f). In addition, more detailed analysis showed that plasma cells and plasmablasts display significantly higher CD38 surface expression levels compared to naïve B cells and all memory B cell subsets, measured by mean fluorescence intensity (MFI) (Fig. 2g). In contrast, CD56^+^CD16^+^ NK cells, CD11c^+^ DC and BDCA2^+^ pDC showed intermediate CD38 expression (Fig. 3a), whereas both naïve T cells and central/effector memory T cells showed very low CD38 expression (Fig. 3b). Consistent with studies in patients with MM [26], about 10% of Treg have high CD38 expression (Fig. 3c and d). The significantly higher CD38 expression on plasma cells and plasmablasts compared to other immune cells suggests the potential for selectively depleting these cells with an anti-CD38 monoclonal therapeutic antibody for treatment of patients with RA and SLE.

### CD38 is expressed predominantly on plasma cells present in synovial biopsies from patients with early RA

**Fig. 4:**
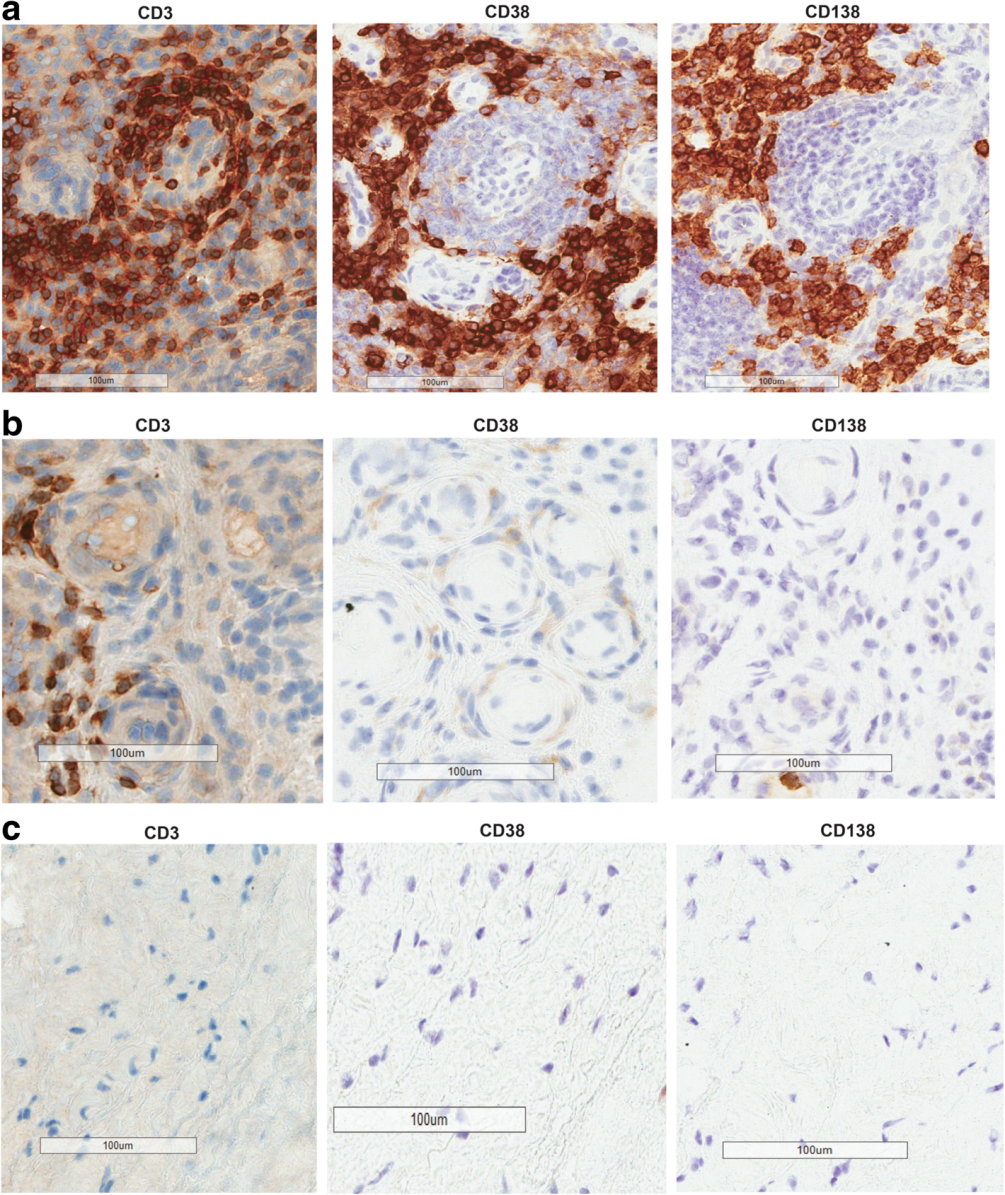
Immunohistochemistry analysis (IHC) of CD38 expression in synovial tissue biopsies from patients with early rheumatoid arthritis (RA). **a** Representative IHC of tissue sections with abundant CD3, CD38 and CD138 staining. **b** Representative IHC of tissue sections with abundant CD3 but sparse CD38 and CD138 staining. **c** Representative IHC of tissue sections with rare CD3, CD38 and CD138 staining. All images are shown at × 20 with 100-μM scale bar in each image. Data shown represent synovial biopsies from nine patients with early RA

To evaluate CD38 protein expression in synovial biopsies from patients with early RA, we performed immunohistochemistry analysis. In treatment-naïve patients with early RA, CD38 stained cells were very abundant in synovial biopsies where inflammatory cell infiltration occurs. As shown in Fig. 3a, CD38 stained cells were observed in the same region as either CD3 stained cells or CD138 stained cells. Synovial samples from five of the nine patients with early RA in our analysis had abundant CD3, CD38 and CD138 stained cells, indicating high T cell and plasma cell infiltration in the synovial biopsies (Fig. 4a and data not shown). Synovial samples from two of the nine patients had abundant T cell staining but few CD38^+^ and CD138^+^ cells (Fig. 4b and data not shown). In these tissues, CD3^+^ T cells were in the same region as cells that displayed lighter CD38 staining than plasma cells. Samples from two patients had very little staining for CD3, CD38 or CD138 (Fig. 4c and data not shown). Samples from these patients did not show immune cell infiltration and may represent a pauci-immune/fibroid pathotype [27]. This finding indicates that plasma cells and T cells were present in synovial tissues from a subpopulation of patients with early RA.

### Daratumumab depletes plasma cells and plasmablasts ex vivo in PBMC from patients with RA and SLE

**Fig. 5:**
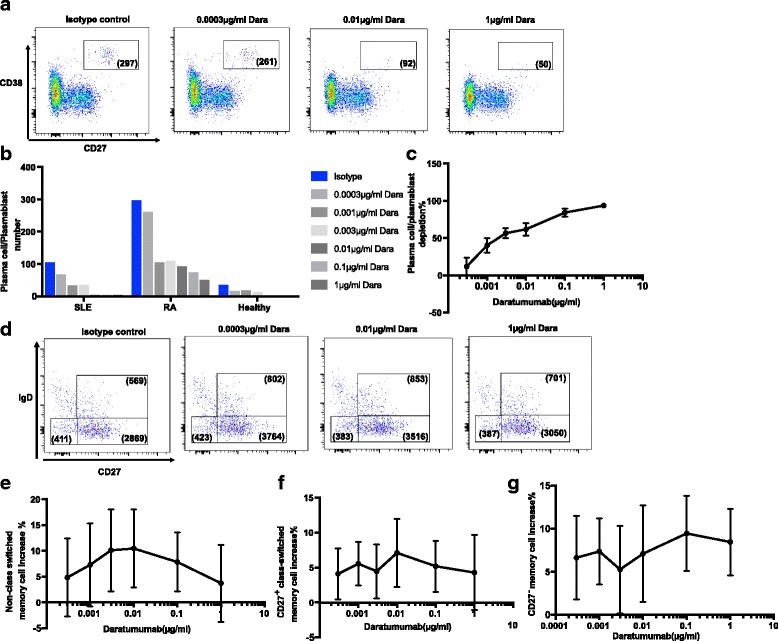
Dose-dependent depletion by daratumumab of plasma cells and plasmablasts from peripheral blood mononuclear cell (PBMC) samples ex vivo. **a** Representative fluorescence-activated cell sorting (FACS) plot of combined plasma cells/plasmablasts. Shown is pre-gated on live singlet CD3^−^CD56^−^CD19^low/mid^CD20^−^ lymphocytes. From left to right, plot shows the representative plasma cell/plasmablast population at 1 μg/ml isotype control, 0.0003 μg/ml daratumumab, 0.01 μg/ml daratumumab and 1 μg/ml daratumumab (Dara), respectively. Number in the quadrant shows absolute number of CD27^hi^CD38^hi^ plasma cells-plasmablasts in each condition at 72 h post-culture. **b** Quantification of plasma cell/plasmablast number at 72 h post-culture with isotype control or daratumumab at indicated concentrations. **c** Dose-response of plasma cell/plasmablast depletion by daratumumab in all donors with systemic lupus erythematosus (SLE) or rheumatoid arthritis (RA) and healthy donors combined. **d** Representative FACS plot of CD27^−^IgD^−^ memory B cell, CD27^+^IgD^−^ class-switched memory B cell and CD27^+^IgD^+^ non-class-switched memory B cell at 72 h post-culture in the presence of daratumumab at each indicated concentration. Number in the quadrant shows absolute number of each memory B cell subset. **e**-**g** Slight increase in non-class-switched memory B cell (**e**), CD27^+^ class-switched memory B cell (**f**) and CD27^−^ memory B cell (**g**) compared to isotype control at each indicated concentration. For each individual donor at each daratumumab concentration, triplicate wells were combined for quantification in **a**, **b** and **d** and then normalized to isotype control in **c** and **e**-**g** Data shown represent four healthy controls, five donors with SLE and four with RA

Daratumumab is a depleting anti-CD38 monoclonal antibody, which has been approved for the treatment of MM [14, 15, 26]. Here we sought to examine if daratumumab would deplete plasma cells and plasmablasts present in PBMCs from patients with SLE and RA, hence providing a rationale for its therapeutic use in plasma cell-dependent autoimmune diseases. Daratumumab has shown complement-dependent cytotoxicity and antibody-dependent cell-mediated cytotoxicity effects in cancer patients [22, 26]. PBMCs from patients with SLE or RA and from healthy donors were incubated with daratumumab at different concentrations for 72 h before FACS analysis. Given the small number of plasma cells and plasmablasts in cell culture, we first combined plasma cells and plasmablasts into one population to gain enough events. Thus, we focused on CD3^−^CD56^−^CD19^low/mid^CD20^−^CD27^hi^CD38^hi^ lymphocytes, which represent both populations as shown in Fig. 5a. Our analysis shows that daratumumab depletes combined plasma cell/plasmablast populations in patients with SLE or RA and in healthy donors in a dose-responsive manner (Fig. 5a-c). In addition, we specifically focused on plasma cells alone using CD3^−^CD56^−^CD19^low/mid^CD20^−^CD27^hi^CD138^hi^ to avoid any complexity due to daratumumab-induced CD38 down-regulation as reported on MM cells [28]. Data show that daratumumab depletes plasma cells in patients with SLE or RA and in healthy donors in a dose-dependent manner (Additional file 1: Figure S1A-C). Furthermore, CD38 is down-regulated on the remaining plasma cells in a dose-dependent manner. The highest concentration of daratumumab (1 μM) induces CD38 down-regulation by ~ 10-fold ex vivo (Additional file 1: Figure S1D-E), to a similar degree as daratumumab-induced CD38 decrease on MM cells in patients after 10 infusions [28]. Similarly, daratumumab also induced CD38 down-regulation on NK cells (Additional file 1: Figure S1F-G), although NK cell depletion is only observed in some donors bearing a larger number of NK cells in the assay (data not shown). However, daratumumab had no significant dose-dependent impact on memory B cells, including non-class-switched memory B cells, class-switched memory B cells or CD27^−^ memory B cells, except that all memory cell subsets were slightly elevated compared to isotype control, independent of the concentration of daratumumab (Fig. 5d-g). Likewise, no significant depletion or CD38 down-regulation was observed on total CD3^+^ T cells or CD14^+^ monocytes in the same assay (Additional file 2: Figure S2). The data indicate that plasma cell/plasmablast are the major target cells of daratumumab depletion ex vivo.

**Fig. 6:**
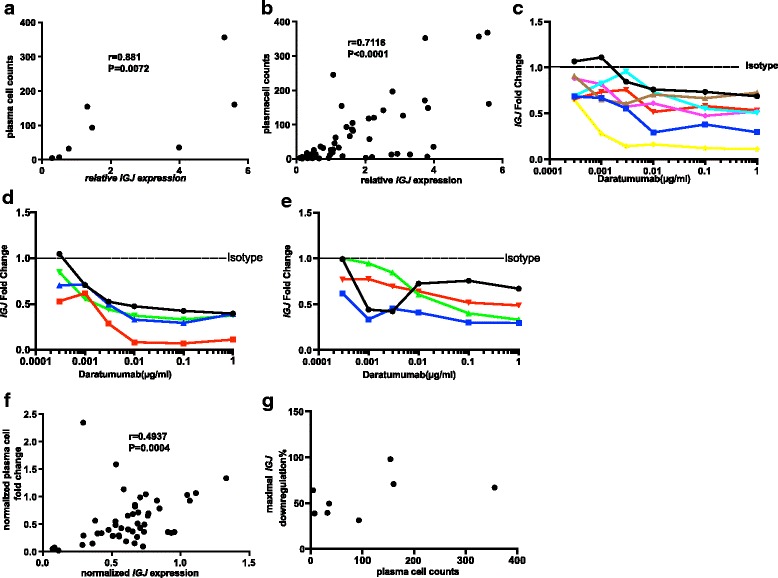
Quantitative PCR analysis shows dose-dependent *IGJ* down-regulation by daratumumab ex vivo. **a** Positive correlation between *IGJ* mRNA and plasma cell count at baseline of isotype control. **b** Positive correlation between *IGJ* mRNA and plasma cell count at different daratumumab concentrations ex vivo. **c-e** *IGJ* expression at different daratumumab concentration at 72 h post-treatment in healthy controls (**c**, *n* = 7) and patients with systemic lupus erythematosus (SLE) (**d**, *n* = 4) and rheumatoid arthritis (RA) (**e**, *n* = 4). Relative expression of *IGJ* in the isotype control was normalized to 1 (shown as dashed line). **f** Positive correlation between normalized *IGJ* mRNA fold change and plasma cell count fold change compared to isotype control at various daratumumab concentrations. **g** Correlation between the number of plasma cells at baseline and the maximal *IGJ* mRNA down-regulation. Data shown in **a**, **b**, **f** and **g** represent two donors with SLE and sixhealthy control donors

IGJ is highly enriched in plasma cells [23]. A previous study showed *IGJ*^hi^ as a biomarker for non-responders to rituximab treatment in patients with RA because these patients have a higher number of plasma cells [29]. Our analysis also shows higher *IGJ* mRNA in synovial biopsies from patients during RA progression (Fig. 1c). We hypothesized that *IGJ* mRNA may serve as a potential pharmacodynamic biomarker for daratumumab treatment. We measured *IGJ* mRNA in the same samples from depletion assays in parallel with FACS as shown above (Fig. 5a-c). As expected, there was positive correlation between the number of plasma cells and *IGJ* mRNA at both baseline (Fig. 6a, isotype control) and in the presence of all daratumumab concentrations tested ex vivo (Fig. 6b). More importantly, analysis showed that daratumumab treatment down-regulates *IGJ* in PBMC samples from healthy donors and from patients with SLE or RA in a dose-responsive manner (Fig. 6c-e). There was positive correlation between the depletion efficacy measured by plasma cell count and *IGJ* mRNA (Fig. 6f), even though there was no significant correlation between the plasma cell count at baseline and the maximal extent of *IGJ* mRNA down-regulation by daratumumab (Fig. 6g). Taken together, our data show that daratumumab effectively depletes plasma cells/plasmablasts in SLE, RA and healthy PBMC samples *ex vivo* and *IGJ* mRNA may serve as a pharmacodynamic biomarker.

## Discussion

CD38 has been extensively studied as a target for the treatment of patients with MM and daratumumab has shown outstanding efficacy in this patient population [14–16]. However, the application of this potential plasma cell/plasmablast-depleting agent such as daratumumab in the treatment of autoimmune disease has not been studied, especially in early stage disease. In this study, using integrative analysis of synovial tissue biopsies obtained from different stages of RA disease and PBMC, we have shown that 1) *CD38* and plasma cell/plasmablast-related genes are up-regulated in ACPA^+^RF^+^ arthralgia and UA disease stages before the onset of RA; 2) CD38 is expressed at the highest level on plasma cells compared to other immune cell populations in RA, SLE and healthy donors in the peripheral blood; and most importantly 3) daratumumab effectively depletes plasma cells/plasmablast in SLE and RA PBMC ex vivo.

Plasma cells and autoantibodies are important in RA pathogenesis as indicated by ACPA production in patients with RA and the correlation between ACPA titer and disease activity score [30]. Staining for CD38 has also been observed in synovial biopsies from patients with established RA [31]. However, there has been no focus on subjects with pre-RA disease (arthralgia and UA) and treatment-naïve patients with early RA. Herein, we showed that the expression of major genes related to plasma cells and plasmablast development and survival is significantly heightened in synovial biopsies from donors with arthralgia and UA compared to healthy donors and those with OA. These include *CD38*, *XBP1*, *IGJ* and *TNFSF13B*, *IRF4* and *PRDM1*, and their up-regulation is observed in synovial biopsies from donors with UA, early RA and established RA. These data indicate that even before the onset of clinically classifiable RA disease, there may be autoantibody-producing plasma cells in the joints, which may drive the progression of the disease. This is in keeping with previous data showing the presence of autoreactive plasma cells in synovial ectopic lymphoid structures (ELS) driving local production of ACPA antibodies [32]. In addition, in the same paper it was shown that ELS act as self-sustained survival niches for plasma cells, as ELS-positive but not ELS-negative synovial tissue displayed a sustained capacity to produce class-switched ACPA when transplanted into SCID mice in the absence of new cells infiltrating the grafts [32]. Thus, the functional presence of plasmablasts/cells in synovial niches together with the high expression of genes involved in their development and survival indicates that therapeutic targeting of antibody-producing plasma cells/plasmablasts with these molecules, in particular CD38, could be a viable strategy for disease intervention in patients with arthralgia to prevent progression to established RA. This notion is further supported by recent observations demonstrating increased levels of circulating IgA plasmablasts in seropositive (ACPA^+^) subjects without any clinical signs of disease [33]. The absence of *IRF4* and *PRDM1* up-regulation in the joints of patients with arthralgia may indicate three possibilities. First, there are not yet enough plasma cells in the joints of these patients to allow for the detection of these changes in transcription factor expression compared to healthy controls. Second, modest changes in these master transcription factors may indeed reflect a decent number of plasma cells/plasmablasts to initiate the disease progression. Third, at this early stage of the disease progression, plasma cells/plasmablasts may migrate into the joints from the periphery and de novo differentiation/development has not yet occurred. Further studies are warranted to determine if plasma cells/plasmablasts are the earliest detectable immune cells in the joints of arthralgia patients and justify the clinical testing of CD38-depleting agents in this patient population.

The above reported IHC results indicate that plasma cells and T cells are dominant in the synovial tissues of treatment-naïve patients with early RA. The data also show that CD38 staining is in the same region as cells that are neither T cells nor plasma cells, which could be macrophages. Therefore, CD38-targeted therapeutics may decrease other pathogenic cells in the synovial tissue while preferentially depleting plasma cells. More careful monitoring of clinical data is warranted to determine the target immune populations in patients with RA or SLE.

We have also shown that plasma cells and plasmablasts can be effectively depleted ex vivo in PBMC samples from donors with SLE or RA and healthy donors. This provides the rationale for depleting the plasma cells/plasmablasts specifically in patients with RA and SLE. NK cells have been reported to decrease significantly in patients with cancer treated with daratumumab [34]. Although it is possible that NK cells become depleted in patients with RA, we saw more robust plasma cell/plasmablast depletion ex vivo in donors with SLE or RA and healthy donors. Therefore, we may be able to find a dose of daratumumab, potentially much lower than the clinical dose in MM, which selectively depletes plasma cells/plasmablasts without significantly affecting NK cells in the same patient. The remarkable decrease in plasma cells and plasmablasts in peripheral blood may serve as a surrogate biomarker for the pharmacodynamic efficacy measurement. Furthermore, the down-regulation of *IGJ* mRNA confirms the depletion of plasma cells upon daratumumab treatment. The dose-response with daratumumab shown in donor PBMCs suggests *IGJ* as a potential surrogate biomarker for plasma cell depletion. Consistent with previous studies showing *IGJ* as a hallmark gene for plasma cells [23], we observed positive correlation between the number of plasma cells and *IGJ* mRNA in all conditions ex vivo (Fig. 6a and b). The positive correlation between the changes in plasma cell number and change in *IGJ* mRNA reinforces the notion of using *IGJ* mRNA as a pharmacodynamic biomarker for daratumumab (Fig. 6f). The donors with more plasma cells at baseline and thus higher *IGJ* mRNA had more readily measurable dose response to daratumumab-mediated depletion ex vivo than the donors with fewer plasma cells and lower *IGJ* mRNA (Figs. 5 and 6, and data not shown). However, the lack of correlation between the number of plasma cells and maximal *IGJ* mRNA down-regulation level may be due to the high antibody-dependent cellular cytotoxicity (ADCC) activity of daratumumab and the small number of cells*,* and therefore very efficient depletion in all donors ex vivo. Nevertheless, the dose response suggests that *IGJ* mRNA level may also serve as a patient stratification marker as we enter the era of personalized medicine, i.e. only patients with higher *IGJ* mRNA and thus a more plasma cells may be treated with daratumumab or other anti-CD38 therapeutic antibodies. The application of *IGJ* as biomarker can potentially facilitate clinical development of anti-CD38 in autoimmune diseases. This is consistent with the finding that identified *IGJ*^hi^ as a biomarker in patients with RA who may not benefit from B cell depletion therapy with rituximab [29]. Plasma cell depleting agents such as daratumumab may prove efficacious in this patient population.

Given the high unmet need for deeper and long-lasting responses in RA and SLE, it is important to develop therapies with more specificity for patient subpopulations based upon selective dysregulated pathways. Patient stratification based on plasma cell and plasmablast analysis may prove more efficacious in clinical testing of anti-CD38 therapeutic agents in RA and SLE. Studies are warranted to determine if daratumumab or other anti-CD38 antibody-based therapies have efficacy in pre-disease states, such as arthralgia and UA, by delaying or preventing the clinical course of disease progression. In summary, our results indicate that plasma cell/plasmablast depleting mechanisms can be used not only for the treatment of established RA but also for the prevention of the disease progression to RA. Furthermore, a plasma cell/plasmablast depleting agent, such as daratumumab, may also show efficacy in other autoantibody-dependent indications, like SLE, IgG4-related disease, multiple sclerosis, myasthenia gravis, primary Sjogren’s syndrome and so on. Finally, in a manner analogous to pre-RA disease, SLE is also considered to have a natural course of evolution with pre-disease state(s) exhibiting asymptomatic autoimmunity [35]. An anti-CD38 or other plasma cell/plasmablast targeting agent may also prevent/delay the progression of asymptomatic autoimmunity to a clinically classifiable lupus stage.

## Conclusions

In conclusion, using integrative analysis of synovial tissue biopsies and PBMC, we have shown that (1) *CD38* and plasma cell/plasmablast-related genes are up-regulated in ACPA^+^RF^+^ arthralgia and UA disease stages before the onset of RA; (2) CD38 is expressed at the highest level on plasma cells in the peripheral blood compared to other immune cell populations in donors with RA or SLE and healthy donors; (3) daratumumab, an approved cancer drug, effectively depletes plasma cells/plasmablasts in SLE and RA PBMC ex vivo. The data suggest that daratumumab and other CD38-targeting therapeutic antibodies may prove efficacious in prevention at the pre-disease stage and in the treatment of plasma-cell-rich established RA and SLE.

## Additional files

Additional file 1: **Figure S1.** Dose-dependent ex vivo depletion of plasma cells by daratumumab in PBMC samples. (A) Representative FACS plot of combined plasma cells. Pre-gated on live singlet CD3^−^CD56^−^CD19^low/mid^CD20^−^lymphocytes. From left to right, plot shows the representative plasma cell population (CD27^hi^CD138^+^) at 1 μg/ml isotype control, 0.0003 μg/ml daratumumab, 0.01μg/ml daratumumab and 1μg/ml daratumumab, respectively. Number in the quadrant shows the absolute number of plasma cells at each condition. (B) Quantification of plasma cells at 72 h post-culture with isotype control or daratumumab (Dara) at indicated concentrations. (C) Dose-response of plasma cell depletion by daratumumab in combined samples from patients with SLE or RA and healthy controls. (D) Representative quantification of CD38 MFI on remaining plasma cells at 72 h post-culture with isotype control or daratumumab at indicated concentrations. (E) Dose-response of CD38 down-regulation on plasma cells by daratumumab in all samples combined as in C-D. For each individual donor at each daratumumab concentration, triplicate wells were combined for quantification in B and D and then normalized to isotype control in C and E. (F) Representative quantification of CD38 MFI on CD56^+^CD16^+^ NK cells at 72 h post-culture with isotype control or daratumumab at indicated concentrations. (G) Dose response of CD38 MFI down-regulation on NK cells by daratumumab in patients with SLE or RA and healthy controls combined. Data shown represent four patients with SLE, four with RA and four healthy controls. (PDF 401 kb)

Additional file 2: **Figure S2.** Daratumumab has no impact on T cells and monocytes ex vivo. (A) Total number of CD3^+^ T cells in each daratumumab concentration at 72 h post-treatment. (B) Quantification of CD38 MFI on CD3^+^ T cells at 72 h post-culture with isotype control or daratumumab at indicated concentrations. (C) Total number of CD14^+^ monocytes in each daratumumab concentration at 72 h post-treatment. (D) Quantification of CD38 MFI on CD14^+^ monocytes at 72 h post-culture with isotype control or daratumumab at indicated concentrations. Data shown represent four patients with SLE, six with RA and six healthy control donors. (PNG 2127 kb)

## Abbreviations

Ab: Antibody
ACPA: Anti-citrullinated protein antibodies
BAFF: B-cell activating factor
bDNA: branched DNA
bp: Base pairs
CRP: C-reactive protein
DMARDs: Disease-modifying anti-rheumatic drugs
ELS: Ectopic lymphoid structures
FACS: Fluorescence-activated cell sorting
FC: Fold change
GEO: Gene Expression Omnibus
IgJ: Immunoglobulin J
IHC: Immunohistochemistry analysis
IRF4: Interferon regulatory factor 4
MM: Multiple myeloma
mRNA: Messenger RNA
NK: Natural killer
OA: Osteoarthritis
PBS: Phosphate-buffered saline
pDCs: Plasmacytoid dendritic cells
PRDM1: PR domain zinc finger protein 1
RA: Rheumatoid arthritis
RF: Rheumatoid factor
RNA-Seq: RNA sequencing
SLE: Systemic lupus erythematosus
TNFα: Tumor necrosis factor alpha
UA: Undifferentiated arthritis
XBP1: X-box binding protein 1
