# Supplementary figures and images for: Integrative analysis reveals CD38 as a therapeutic target for plasma cell-rich pre-disease and established rheumatoid arthritis and systemic lupus erythematosus
Source: Arthritis Res Ther. 2018 May 2;20:85. doi: 10.1186/s13075-018-1578-z (PMC5932888; doi:10.1186/s13075-018-1578-z)

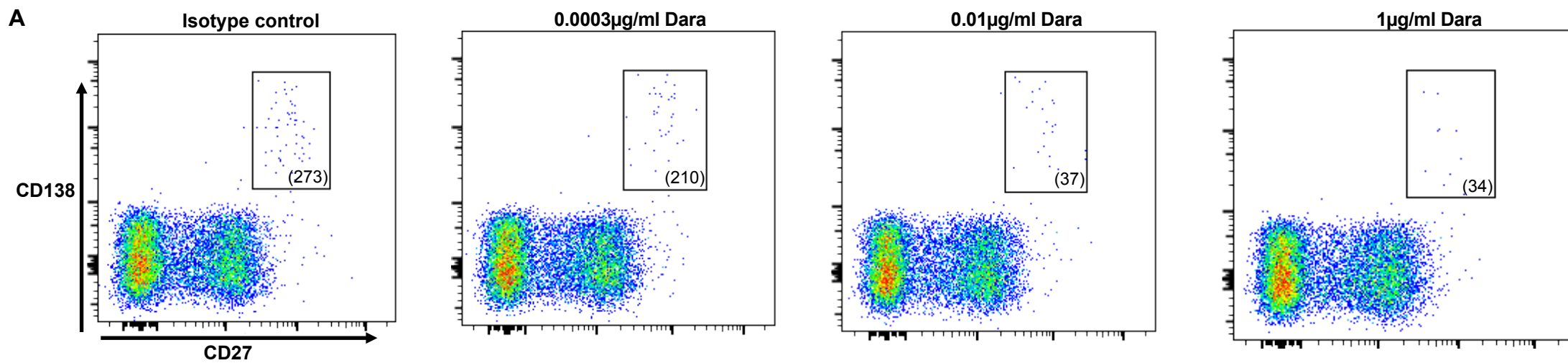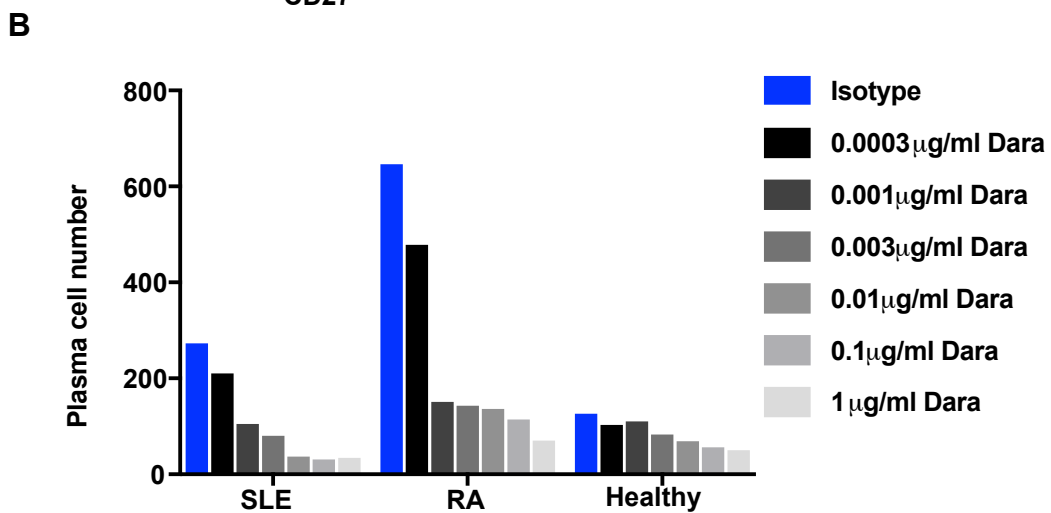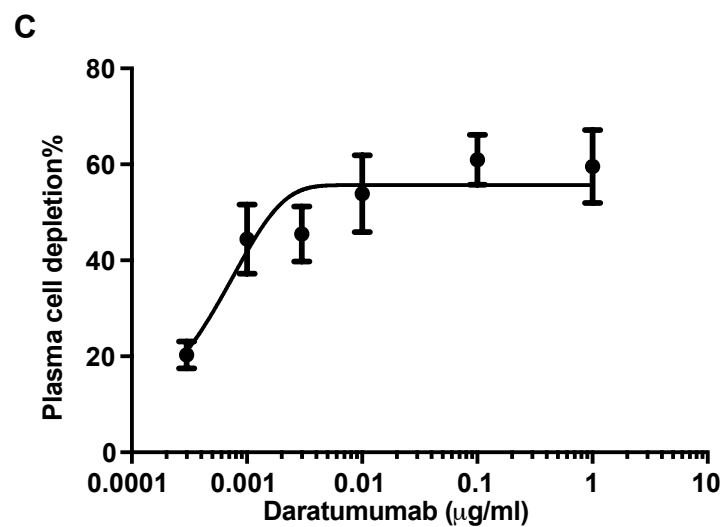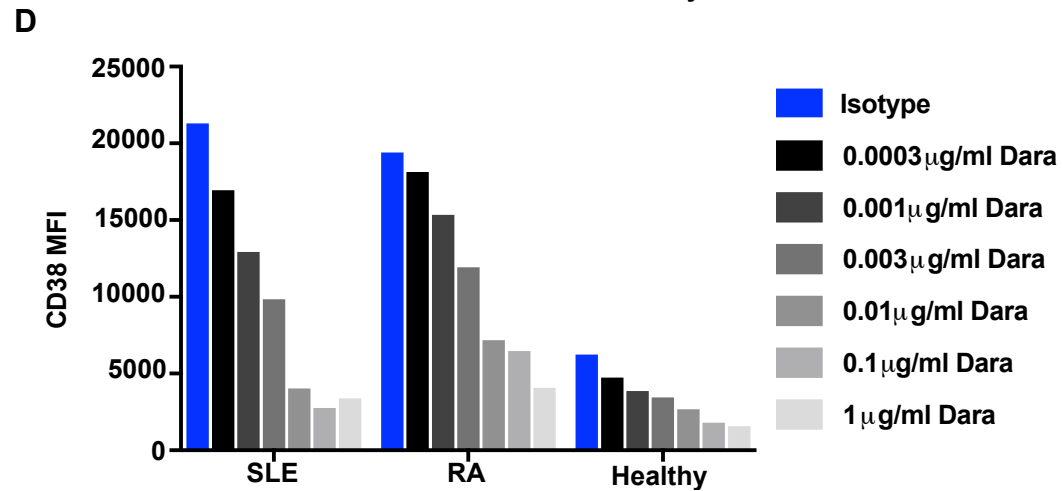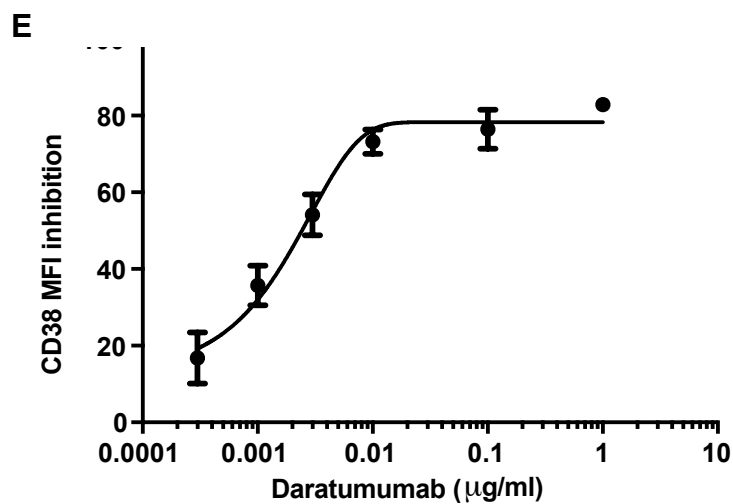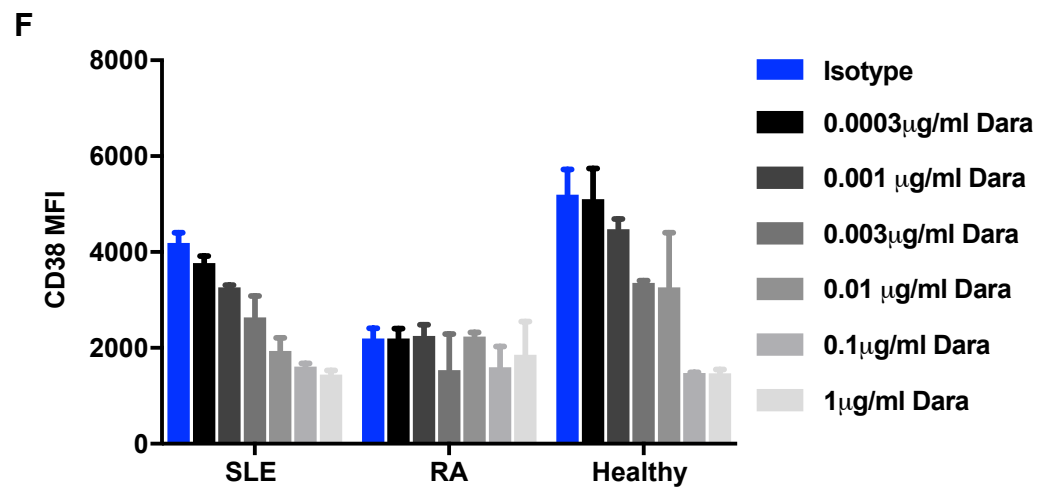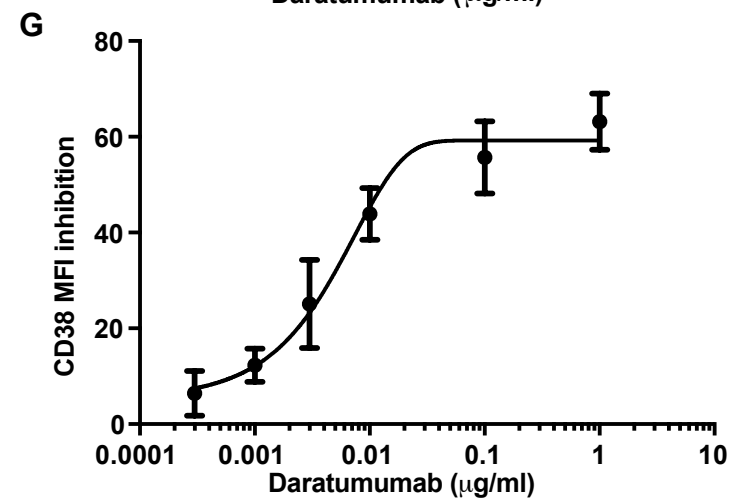

Supplement: Supplementary file 1 — Figure S1. Dose-dependent ex vivo depletion of plasma cells by daratumumab in PBMC samples. (A) Representative FACS plot of combined plasma cells. Pre-gated on live singlet CD3−CD56−CD19low/midCD20−lymphocytes. From left to right, plot shows the representative plasma cell population (CD27hiCD138+) at 1 μg/ml isotype control, 0.0003 μg/ml daratumumab, 0.01μg/ml daratumumab and 1μg/ml daratumumab, respectively. Number in the quadrant shows the absolute number of plasma cells at each condition. (B) Quantification of plasma cells at 72 h post-culture with isotype control or daratumumab (Dara) at indicated concentrations. (C) Dose-response of plasma cell depletion by daratumumab in combined samples from patients with SLE or RA and healthy controls. (D) Representative quantification of CD38 MFI on remaining plasma cells at 72 h post-culture with isotype control or daratumumab at indicated concentrations. (E) Dose-response of CD38 down-regulation on plasma cells by daratumumab in all samples combined as in C-D. For each individual donor at each daratumumab concentration, triplicate wells were combined for quantification in B and D and then normalized to isotype control in C and E. (F) Representative quantification of CD38 MFI on CD56+CD16+ NK cells at 72 h post-culture with isotype control or daratumumab at indicated concentrations. (G) Dose response of CD38 MFI down-regulation on NK cells by daratumumab in patients with SLE or RA and healthy controls combined. Data shown represent four patients with SLE, four with RA and four healthy controls. (PDF 401 kb) [file 13075_2018_1578_MOESM1_ESM.pdf]

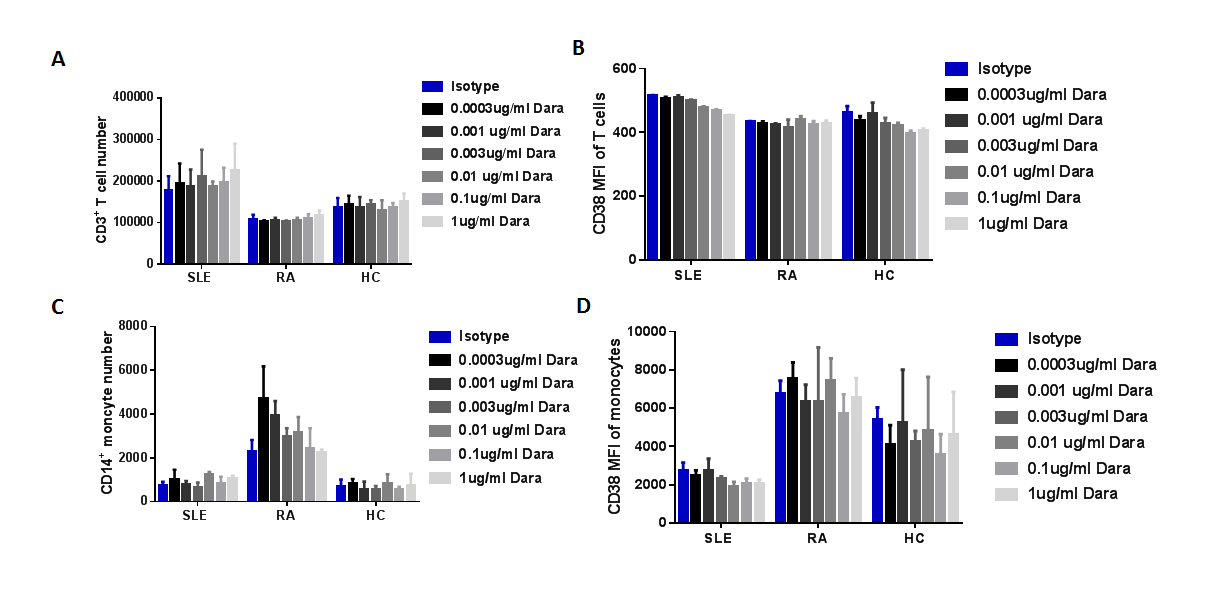

Supplement: Supplementary file 2 — Figure S2. Daratumumab has no impact on T cells and monocytes ex vivo. (A) Total number of CD3+ T cells in each daratumumab concentration at 72 h post-treatment. (B) Quantification of CD38 MFI on CD3+ T cells at 72 h post-culture with isotype control or daratumumab at indicated concentrations. (C) Total number of CD14+ monocytes in each daratumumab concentration at 72 h post-treatment. (D) Quantification of CD38 MFI on CD14+ monocytes at 72 h post-culture with isotype control or daratumumab at indicated concentrations. Data shown represent four patients with SLE, six with RA and six healthy control donors. (PNG 2127 kb) [file 13075_2018_1578_MOESM2_ESM.png]
